# Sero-prevalence and risk factors for hepatitis E virus infection among pregnant women in the Cape Coast Metropolis, Ghana

**DOI:** 10.1371/journal.pone.0191685

**Published:** 2018-01-25

**Authors:** Dorcas Obiri-Yeboah, Yaw Asante Awuku, Joseph Adu, Faustina Pappoe, Evans Obboh, Paul Nsiah, Daniel Amoako-Sakyi, Jacques Simpore

**Affiliations:** 1 Department of Microbiology and Immunology, School of Medical Sciences, University of Cape Coast, Cape Coast, Ghana; 2 Department of Internal Medicine and Therapeutics, School of Medical Sciences, University of Cape Coast, Cape Coast, Ghana; 3 Department of Obstetrics and Gynecology, School of Medical Sciences, University of Cape Coast, Cape Coast, Ghana; 4 Department of Chemical Pathology, School of Medical Sciences, University of Cape Coast, Cape Coast, Ghana; 5 Laboratory of Molecular Biology and Genetics (LABIOGENE), University of Ouagadougou, Ouagadougou, Burkina Faso; University of Cincinnati College of Medicine, UNITED STATES

## Abstract

**Background:**

Hepatitis E virus is an emerging infection in Africa with poor maternal and foetal outcomes. There is scanty data on the sero-prevalence of HEV infection among pregnant women in Ghana. This study highlighted the prevalence and risk factors associated with HEV infection among pregnant women in Cape Coast Metropolis, Central Region of Ghana.

**Methods:**

A multicenter (3 selected sites) analytical cross sectional study involving 398 pregnant women in the Cape Coast metropolis was conducted. HEV (Anti-HEV IgG and Anti-HEV IgM) ELISA was performed. Sero-positive women had liver chemistries done and data collected on maternal and neonatal outcomes. Data analyses were performed using Stata version 13 software (STATA Corp, Texas USA).

**Results:**

Mean age was 28.01 (± 5.93) years. HEV sero-prevalence was 12.2% (n = 48) for IgG and 0.2% (n = 1) for IgM with overall of 12.3%. The odds of being HEV sero-positive for women aged 26–35 years was 3.1 (95% CI: 1.1–8.1), p = 0.02 and ≥36 years it was 10.7 (95% CI; 3.4–33.5), p = 0.0001. Living in urban settlement was associated with lowest odds of HEV infection {OR 0.4 (95% CI; 0.2–0.8), p = 0.01}. Factors with no statistical evidence of association include main source of drinking water and history of blood transfusion. The sero-prevalence of HEV IgG increased progressively across trimesters with the highest among women in their third trimester (55.3%). None of the 49 HEV sero-positive women had elevated ALT level. Ten (N = 41) of the neonates born to sero-positive women developed jaundice in the neonatal period. The mean birth weight was 3.1kg (SD 0.4).

**Conclusion:**

HEV sero-prevalence among pregnant women in the Cape Coast Metropolis is high enough to deserve more attention than it has received so far. It is therefore important to conduct further research on the potential impact on maternal and neonatal mortality and morbidity in Ghana.

## Introduction

Hepatitis E virus (HEV) was first discovered through retrospective testing of stored sera from the 1955–56 outbreak in New Delhi, India. It was initially thought to be Hepatitis A virus however, testing revealed a novel non-enveloped single stranded RNA virus, which was subsequently named HEV [1–3]. HEV is endemic in resource poor regions of the world and most prevalent in low and middle-income countries (LIMCs) characterized by poor sanitation conditions [4, 5]. Hence, HEV infection is commonly acquired through consumption of faecally contaminated water [6–8]. Transmission via rodents and internal organs of pigs has also been reported, occurring more frequently in industrialized countries [7, 9].

Current estimates suggests that about one-third of the world’s population have been infected with HEV and over 20 million HEV infections occur globally each year. However, infection is usually silent and thus, out of the 20 million estimated cases only 3 million develop symptoms [5, 8]. The disease is usually mild in men and non-pregnant women [10, 11]. Also, a small proportion of infected people of the general population, develop fulminant hepatitis with a case fatality rate of 2% [5]. However, high fatality rate, as high as 40% is seen among pregnant women [10, 12, 13]. Bonney *et al* [14] found HEV to be associated with mortality in their case series from Accra Ghana. Additionally, severe HEV associated complications such as coagulation defects, fulminant hepatic failure (FHF), preterm labor, and postpartum haemorrhage are reported to be common among pregnant women in their third trimester [15–17]. Hence, HEV is a major global health problem with a disproportionately high disease burden among pregnant women. Despite the fact that HEV could have dire consequencies for maternal and infant health in LIMCs, data on the epidemiolgy of HEV in Ghana is limited [18].

Like most LIMCs, Ghana has embarked on a lot of programs to improve maternal and child health indices. Some of these programs have yielded positive results but serious deficits persist. Considering that HEV infection is most prevalent and dangerous among pregnant women, it is imperative to access the contribution of HEV to poor pregnancy outcomes in Ghana. This study highlights the prevalence and risk factors associated with HEV infection among pregnant women in the Cape Coast Metropolis of the Central Region of Ghana.

## Methodology

### Ethical considerations

Ethical approval was obtained from Institutional Review Boards of UCC. Study participants were given study information after which they signed or thump printed written informed consent indicating consent to participate in the study.

### Study design and subjects

An analytical cross-sectional study was conducted in three (3) selected antenatal clinics (Cape Coast Teaching Hospital (CCTH), Cape Coast Metropolitan Hospital and Ewim Health Centre) in the Cape Coast Metropolis, Central Region. These 3 facilities have the heaviest antenatal clinic (ANC) attendance per day and receive women across the Metropolis. A systematic sampling method was used at each ANC daily to recruit maximum of 20 eligible (18 years and above with no obstetric complications) pregnant women per day. After recruitment, the study protocol was explained to each woman and written informed consent was obtained. Women who consented to participate were administered a pre-tested questionnaire to obtain information on relevant socio-demographic and medical/obstetric characteristics. This questionnaire had been pre-tested using 10 pregnant women from the CCTH. It was administered both in English and the Fante language and feedback from this exercise was then used to finalize the questionnaire. In addition, arrangements were made to be able to follow up HEV sero-positive women and get information on events after recruitment till the end of the neonatal period.

### Sample collection and processing

Blood samples (5mls) were collected from the antecubital vein into EDTA vacutainer tubes and transported to the School of Medical Sciences (SMS) laboratory at the University of Cape Coast (UCC). The sample was separated by centrifugation and kept at -20°C until used.

HEV (Anti-HEV IgG and Anti-HEV IgM) ELISA was performed on all samples in duplicate using INNOVITA kit (Tangshan, China), following the manufacturer’s instructions. The sensitivity of this assay is 94.36% and the specificity is 97.19% according to the manufacturer’s manual. In brief, the wells of the microcroplate were coated with the recombinant HEV antigen expressed by genetic engineering. Mouse anti-human IgM antibody labeled with enzyme was used as tracer and tetramethyl benzidine as a chromogenic system. The plasma was added into the wells of the microplate first. Samples containing specific IgM or IgG antibodies against HEV reacted with the antigen in the microplate sealer and formed an antibody-antigen complex in the wells, the complex reacted with the enzyme-labeled mouse anti-human IgM or IgG antibody and further formed antigen-antibody-enzyme-labeled-antibody complex. Excess unbound substances were removed by washing after which the substrate and chromogenic agent was added and incubated at 37°C for 30minutes. The substrate added was oxidized to blue under the action of the enzyme. The reaction system became yellow after addition of the stop solution and the optical density of the color, expressed in “OD” value, was measured at a specific wavelength (450nm). Readings were done within 10minutes after termination of the reaction. In each plate, two negative controls and two positive controls were included for standardization and validation of results. The presence or absence of anti-HEV IgM or IgG antibody was determined by relating the absorbance of the specimen to the cut-off value, which is the mean absorbance of the negative control plus 0.10. The presence or absence of HEV IgM or IgG in the sample was determined by the color intensity. Wells containing samples negative were colorless. Specimens giving a value less than the Cut-off value were considered negative for this assay.

For women who were positive for either IgG or IgM, their liver chemistries were determined. Serum ALT, AST, GGT, total and direct bilirubin were assayed using ChemWell 2910 auto-analyzer (Awareness Technologies, USA) according to manufacturer’s protocol.

### Statistical analysis

Sample size estimation was done using the highest possible population of pregnant women in the study area, 95% confidence level and 5% margin of error the highest sample size calculated was 383. Data analyses were performed using Stata version 13 software (STATA Corp, Texas USA). Data distribution of the various variables was determined to enable the application of parametric or non-parametric statistics where appropriate. Descriptive analysis of socio-demographic, behaviour and other relevant characteristics of the study population were done using appropriate measures of central tendencies. Between-group comparisons were done using means, median, or frequencies where appropriate and differences were deemed statistically significant at *p > 0*.*05*. Bivariate and multivariate analysis was done for HEV prevalence and associations are presented as odds ratios (OR) with 95% confidence intervals (CI). Variables with *P*-values ≤0.20 and a priori factors like age were put in the model for multivariate analysis.

## Results

A total of 400 pregnant women were recruited in this study and 398 pregnant had valid HEV ELISA results (IgG and/or IgM) and hence included in the analysis. The mean age was 28.01 (± 5.93) years. Majority of the study subjects (59.5%) were married. The predominant religion was Christianity (92.0%). Majority (63.8%) drank pipe borne water directly while 35.9% mainly drank sold sachet water. Those from the rural areas were 57 (14.3%) with peri-urban and urban being 156 (39.2%) and 185 (46.5%) respectively (Table 1).

**Table 1:** Socio-demographic characteristics of study participants (N = 398).

*Variable*	*Mean (SD)/Median (IQR) /n (%)*
***Age(years) N = 393***	
*Mean*	28.0 (±5.9)
*<25*	142 (36.1)
*26–35*	205 (52.2)
*>36*	46 (11.7)
***Religion***	
*Christians*	366 (92.0)
*Moslems*	31 (7.8)
*Others*	1 (0.3)
***Marital status***	
*Single*	89 (22.4)
*Married*	242 (60.8)
*Cohabiting*	67 (16.8)
***Educational Status***	
*None to primary level*	101 (25.4)
*Secondary (Senior high)level*	247 (62.1)
*Tertiary level*	50 (12.6)
***Employment***	
*Unemployed*	78 (19.6)
*Unskilled employment*	275 (69.1)
*Skilled employment*	45 (11.3)
***Place or Residence***	
*Rural*	57 (14.3)
*Peri-urban*	156 (39.2)
*Urban*	185 (46.5)
***Source of drinking water***	
*Pipe borne water*	254 (63.8)
*Sachet water*	143 (35.9)
*Stream*	1 (0.3)

The median number of previous pregnancies among study participants was 2 (1, 3) with 24.9% (n = 97) and 0.5% (n = 2) having has 1–2 and ≥3 spontaneous abortions respectively. Fourteen (3.7%) and 41 (10.6%) had history of 1 and 2 previous stillbirths respectively. As much as 39.7% (n = 155) had not tested for HIV infection in this pregnancy. The mean gestational age at recruitment was 25.9 (±8.9) with majority being in their third trimester (183, 47.2%). Eleven (2.8%) of the participants had history of jaundice in this pregnancy. The HEV sero-prevalence among these pregnant women was 12.2% (n = 48) for IgG and 0.2% (n = 1) for IgM (Table 2). The overall HEV sero-prevalence was 12.3%.

**Table 2:** Obstetric and other relevant parameters of study participants.

*Variable*	*Mean (SD)/Median (IQR) /n (%)*
***Number of pregnancies in the past (N = 393)***	
*Median*	2 (1,3)
*0–4*	369 (93.9)
*≥5*	24 (6.1)
***Number of spontaneous abortions (N = 389)***	
*0*	290 (74.6)
*1–2*	97 (24.9)
*≥3*	2 (0.5)
***Number of past stillbirths (N = 384)***	
*0*	329 (85.7)
*1*	41 (10.6)
*2*	14 (3.7)
***Number of children (N = 398)***	
*Median*	1 (0, 2)
*0*	115 (28.9)
*1–4*	273 (68.6)
*≥5*	10 (2.5)
***HIV status (N = 390)***	
*Positive*	1 (0.3)
*Negative*	234 (60.0)
*Don’t know*	155 (39.7)
***History blood transfusion (N = 398)***	
*Yes*	31 (7.8)
*No*	364 (91.4)
*Don’t know*	3 (0.8)
***Gestational age of current pregnancy at recruitment (N = 387)***	
*Mean*	25.9 (±8.9)
*First trimester*	30 (7.8)
*Second trimester*	174 (45.0)
*Third trimester*	183 (47.2)
***History of jaundice in current pregnancy (N = 398)***	
*Yes*	11 (2.8)
*No*	387 (97.2)
***HEV IgG (N = 395)***	
*Positive*	48 (12.2)
*Negative*	347 (87.8)
***HEV IgM (N = 395)***	
*Positive*	1 (0.2)
*Negative*	394 (99.8)

### Risk factor analysis

Relative to women below 26 years, the odds of being HEV positive was higher in those aged between 26 and 35 years {OR 3.1 (95% CI: 1.1–8.1) p = 0.02} and those at or more than 36 years {OR 10.7 (95% CI; 3.4–33.5), p = 0.0001}. Living in urban settlement was associated with the lowest odds of HEV infection on bivariate analysis {OR 0.4 (95% CI; 0.2–0.8), p = 0.01} but not after multivariate analysis, p = 0.27. Pregnant women who did not know their HIV status in this pregnancy had higher odds of HEV infection compared with those who tested negative {aOR 2.2 (95% CI; 1.1–4.5), p = 0.03}. Factors which did not show statistical evidence of an association include marital and employment status, main source of drinking water, educational level and history of blood transfusion (Table 3).

**Table 3:** Factors associated with HEV IgG positivity among study participants.

*Variable*	*OR (95% CI)*	*P-value*	*aOR (95% CI)*	*P-value*
***Age (years)***				
*<25*	-		-	
*26–35*	2.1 (0.9–4.9)	0.07	3.1 (1.1–8.1)	***0*.*02***
*>36*	5.8 (2.1–16.2)	***0*.*0001***	10.7(3.4–33.5)	***0*.*0001***
***Marital status***				
*Single*	-		-	
*Married*	0.9 (0.4–1.8)	0.78	0.5 (0.2–1.1)	0.09
*Cohabiting*	0.2 (0.1–1.0)	***0*.*04***	0.3 (0.1–1.2)	0.09
***Place or Residence***				
*Rural*	-		-	
*Peri-urban*	0.5 (0.2–1.1)	0.09	0.9 (0.3–2.5)	0.89
*Urban*	0.4 (0.2–0.8)	***0*.*01***	0.5 (0.2–1.5)	0.27
***Number of past stillbirths***				
*0*	-		-	
*≥1*	1.6 (0.9–3.1)	0.14	1.3 (0.5–3.1)	0.56
***HIV status***				
*Positive*				
*Negative*	-		-	
*Don’t know*	2.5 (1.3–4.6)	***0*.*004***	2.2 (1.1–4.5)	***0*.*03***
***Educational Status***				
*None to primary level*	-			
*Secondary(Senior high) level*	0.9 (0.5–1.9)	0.97		
*Tertiary level*	0.5 (0.2–1.9)	0.37		
***Employment***				
*Unemployed*	-			
*Unskilled employment*	1.8 (0.7–4.5)	0.18		
*Skilled employment*	1.8 (0.5–6.2)	0.31		
***Main source of drinking water***				
*Pipe borne water*	-			
*Sachet water*	0.7 (0.4–1.4)	0.29		
*Stream*				
***History blood transfusion***				
*Yes*	-			
*No*	2.1 (0.5–9.2)	0.31		
*Don’t know*				

**Fig 1:**
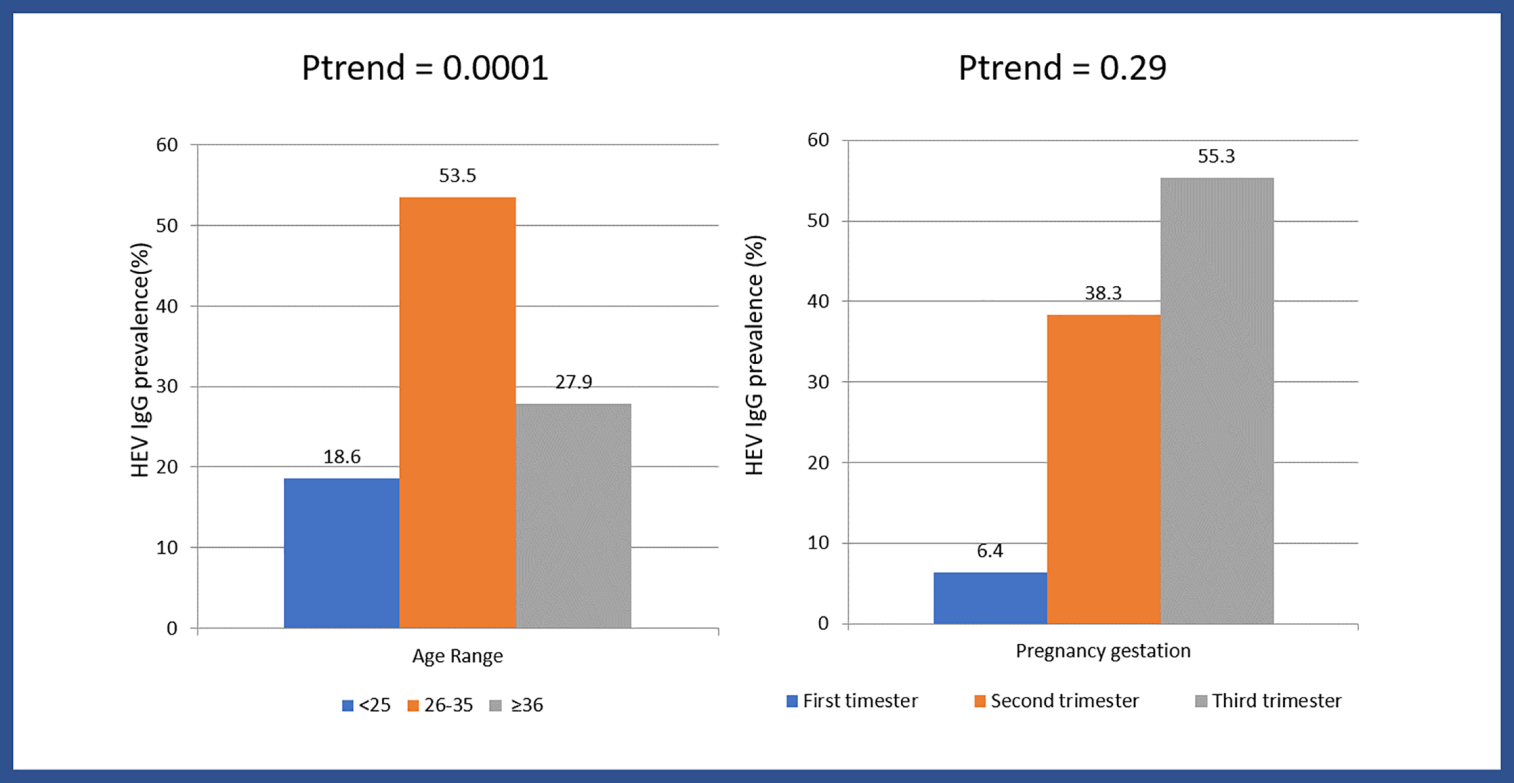
HEV IgG distribution by maternal age and estimated gestational age among positive pregnant women (N = 48).

The highest HEV IgG positivity was detected among women in the 26–35 years age range (53.5%) and lowest among age group ≤25 years (18.6%), ptrend = 0.0001. The prevalence of HEV IgG increased progressively across the trimester of pregnancy with the highest among women in their third trimester (55.3%), Fig 1.

### Pregnancy and neonatal outcomes

For Pregnant women who were positive for IgG and/or IGM, none had abnormal values for ALT, GGT, albumin, and indirect bilirubin levels. Six (12.2%) had elevated AST levels while 2 (4.1%) had raised direct bilirubin level (Table 4).

**Table 4:** Liver function results of 49 pregnant women who were positive for HEV IgG and/or IgM.

Variable	n (%)
**AST levels, U/l**	
*Normal values (5–34)*	43 (87.8)
*High values*	6 (12.2)
**ALT, U/l**	
*Normal values (40–50)*	49 (100.0)
*High values*	0 (0.0)
**GGT, U/l**	
*Normal values (9–36)*	49 (100.0)
*High values*	0 (0.0)
**Albumin, g/l**	
*Normal values (34–50)*	49 (100.0)
*Abnormally low values*	0 (0.0)
**Indirect bilirubin, μmol/l**	
*Normal values (1.7–17)*	49 (100.0)
*High values*	0 (0.0)
**Direct bilirubin, μmol/l**	
*Normal values (0–10.3)*	47 (95.9)
*High values*	2 (4.1)

All HEV sero-positive women in this study delivered at term with mean baby birth weight of 3.1kg (SD 0.4). 12.2% (5/41) having birth weight ≤2.5kg. A total of 10 (23.8%) of their neonates developed clinical jaundice but none of them requiring admission (Table 5).

**Table 5:** Pregnancy and neonatal outcome among HEV positive women (N = 42).

*Variable*	*n (%)*
***History of jaundice after recruitment into the study*** ***delivery?***	
*Yes*	0 (0.0)
*No*	42 (100.0)
***Was delivery at term?***	
*Yes*	42 (100.0)
*No*	0 (0.0)
***Any post-delivery complication for the woman?***	
*^#^Yes*	2 (4.8)
*No*	40 (95.2)
***Birth weight (kg), N = 41***	
*Mean (SD, Range)*	3.1 (±0.4, 2.5–4.2)
*≤2.5*	5 (12.2)
*>2.5*	36 (87.8)
***Any neonatal jaundice?***	
*Yes*	10 (23.8)
*No*	32 (76.2)
***Was the baby admitted for management of jaundice? N = 10***	
*Yes*	0 (0.0)
*No*	10 (100.0)

^#^Post-partum hemorrhage

## Discussion

Hepatitis E virus, is emerging as a leading cause of acute viral hepatitis and poor obstetric outcomes [19, 20]. This study showed that the overall sero-prevalence of HEV infection amongst participating pregnant women in the Cape Coast metropolis was 12.3%. This finding is consistent with the result of similar studies done in Burkina Faso (10.6%), and Gabon (14.1%) but lower than the sero-prevalence of the study done in Accra, Ghana by Adjei *et al*, where the seroprevalence of hepatitis E virus in pregnant women was 28.7% [21–23]. It was also lower than HEV infection among pregnant women in Egypt (84.3%), Sudan (41%) and India (33.6%) [24–26]. These differences aligns with the fact of HEV epidemiology, especially, the fact that it is mainly transmitted through faecal contamination of drinking water. Perhaps, these differences reflect that level of sanitation in areas where these studies were conducted. In their systematic review conducted using data published in Ghana so far on HEV infection, the researchers found the overall sero-prevalence to range between 5.8–71.55% [18]. These variations in the prevelence of HEV also provide valuable insight in the formulation of control stratergies, especially the allocation of resources.

Additionally, majority of the infected participants had evidence of past infection (IgG 12.2%) whereas less than 1% had recent infection (IgM: 0.2%). Richard Ofori-Danso and Akosua Adom Agyeman found the IgG and IgM sero-prevalence to range from 0–45.3% and 0.7–45.9% respectively [18]. Humoral immune response play a major role in the course of infectious diseases including HEV infection. Although we could not investiagte mother to child transmission among the participlants, it is possible that congenital cases may or may not occur. In Egypt, El sayed *et al*. screened 29 neonates and their mothers for HEV infection. They found that, 5 neonates were positive for anti HEV-IgG and had HEV viremia same as their mothers. The neonates also had severe HEV associated clinical manifestations. One neonate from another mother with veremia was positive for both HEV IgG and IgM. However, 3 neonates from mothers with positive IgG and viremia were not infected [27].

There appeared to be a direct association between increasing age and increasing positivity indicating that the older individuals have had longer exposure to the external environment thereby exposing them to the virus. This finding is consistent with a study done in Egypt [24] and in Ethiopia [7]. We also found that, the highest prevalence was detected among women in the third trimester of pregnancy and this phenomenon is not fully understood. Similar finding was reported in central India by Shinde *et al* [10].

Latora *et al* indicated in a study conducted in Burkina Faso, that there may a possible significant HEV/HIV coinfection among pregnant women [28]. This study is unable to make inferences on HEV/HIV coinfection since only one of the participants was HIV positive. Feldt *et al* [29] found HEV sero-prevalence as high as 45.3% among 402 HIV positive patients in Ghana. It is worth nothing that a high proportion of pregnant women in this study had no knowledge of their HIV status. This is worrying since the prevention of mother to child transmission of HIV in Ghana requires routine offer of HIV testing to all pregnant women [30]. A study by Caron *et al* also found 7.1% of HEV/HIV coinfection [31]. It has been reported that HIV infected women are at risk for acute or severe infection if they are exposed to HEV during pregnancy [32–35].

Anti-HEV reactivity among pregnant women who lived in rural areas was higher than that of their counterparts who resided at peri-urban and those in the urban areas in this study. This was not surprising as high rates of HEV infection are usually seen in areas with low standards of living where major contamination of water supply is likely to occur. These includes, low standard of hygiene, lack of proper disposal system plus unsafe drinking water supply. Similar findings are reported by Kafando *et el* in their study among pregnant women in Ouagadougou in Burkina Faso [36].

Blood transfusion is a possible route of HEV transmission [37]. This study however did not find an association with blood transfusion. A study in Ghana concluded that HEV may not pose a significant risk to blood transfusion services in Ghana [38].

A total of 11 (2.8%) participants in this study had reported an episode of jaundice in this pregnancy which had resolved by the time of recruitment into this study. Having HEV IgG or IgM (1 participant) antibodies, was not associated with significant liver function derangement. Viral liver injury is usually associated with elevated serum levels of ALT and a number of studies have shown the association between HEV and deranged liver function and possible fulminant hepatitis [12, 14, 39] thus making it important to screen pregnant women with deranged liver function for HEV in addition to usual investigations done.

In this study, the woman who was IgM positive delivered a term baby who developed jaundice in the neonatal period but this resolved without admission. Other studies documented significant poor foetal and neonatal outcomes [10, 17] and in this study, 20 (N = 41) neonates developed jaundice but none of them required admission.

This study had some limitations which include the fact that it was facility based and this could miss out some community level factors affecting pregnant women who do not attend ANC. This limitation is however partly mitigated by the fact that detail information on the residence of study participants were taken to enable us link each study participant with specific neighborhoods in the study area. Another limitation of this study is the lack of molecular viral diagnosis using polymerase chain reaction (PCR) which would have confirm actual rate of HEV infection especially for the IgM positive participant. Meldal *et al* 2013 reported the potential challenges with ELISA testing for HEV and the role molecular assays could play in confirmation particularly acute infection [38]. Despite these, the research provides additional data on this understudied but very relevant topic of HEV infection among pregnant women and will form the basis for further research focusing on molecular diagnostic methods which can also be used to confirm vertical transmission.

In conclusion, this study showed that the HEV sero-prevalence among pregnant women in the Cape Coast Metropolis is high enough to deserve more attention than it has received so far. It is therefore important to conduct further research on the potential impact on maternal and neonatal mortality and morbidity.

## Supporting information

S1 Dataset This is the complete dataset for the manuscript. (XLSX)

S1 Questionnaire This is the English and Fante version of the questionnaire administered to the pregnant women. (DOC)
