# Supplementary material for: Sero-prevalence and risk factors for hepatitis E virus infection among pregnant women in the Cape Coast Metropolis, Ghana
Source: PLoS One. 2018 Jan 25;13(1):e0191685. doi: 10.1371/journal.pone.0191685 (PMC5784989; doi:10.1371/journal.pone.0191685)
Supplement: S1 Questionnaire — (DOC) [file pone.0191685.s002.doc]

**APPENDIX A: QUESTIONNNAIRE**

| **TITLE:**   **Prevalence, Diagnostic options and risk factors for Hepatitis E Virus infection among pregnant women in Cape Coast Metropolis of Ghana.** | | | | |
| --- | --- | --- | --- | --- |
| **DATE OF INTERVIEW:** | | **STUDY CODE *(pre printed)*:** | | **UNIQUE NUMBER:** |
| **NAME OF INTERVIEWER:** | | **SIGNATURE:** | |
| **SOCIO-DEMOGRAPHICS & RISK FACTORS** | | | | |
| **No.** | **QUESTIONS** | **CODE** | | **RESPONSE(S)** |
| **Q1** | **AGE IN YEARS AT LAST BIRTH DATE** | **(*write the actual number down)*** | |  |
| **Q2** | **OCCUPATION** | 1. **Trader** 2. **Civil servant** 3. **Health personnel** 4. **Unskilled labourer** 5. **Unemployed/housewife** 6. **Other** | |  |
| **Q3** | **RELIGION** | 1. **Christian** 2. **Moslem** 3. **Traditional** 4. **Others** | |  |
| **Q4** | **MARITAL STATUS** | 1. **Single** 2. **Married** 3. **Cohabiting** 4. **Divorced** 5. **Widowed** | |  |
| **Q5** | **LEVEL OF EDUCATION** | 1. **No formal education** 2. **Primary** 3. **J.S.S** 4. **Middle school form 4** 5. **Secondary** 6. **Tertiary** | |  |
| **Q6** | **WHERE DO YOU LIVE?** | ***(write the actual town down)*** | |  |
| **Q7** | **WHAT IS YOUR SOURCE OF DRINKING WATER?** | 1. **Pipe borne water** 2. **Sachet** 3. **Stream/River** 4. **Other *(specify)*** | |  |
| **Q8** | **HOW MANY PREGNANCIES HAVE YOU HAD IN THE PAST?** | **(*write the actual number down) If the answer is 0 then skip to Q10*** | |  |
| **Q9** | **HOW MANY OF THESE PREGNANCIES RESULTED IN STILLBIRTH?** | **(*write the actual number down)*** | |  |
| **Q10** | **HOW MANY OF THESE PREGNANCIES RESULTED IN MISCARRIAGE/ABORTION?** | **(*write the actual number down)*** | |  |
|  | **HOW MANY LIVE CHILDREN DO YOU HAVE?** | **(*write the actual number down)*** | |  |
|  | **WHAT IS YOUR HIV STATUS?** | 1. **Positive** 2. **Negative** 3. **Don’t Know** |  | |
|  | **HAVE YOU EVER BEEN GIVEN A BLOOD TRANSFUSION?** | 1. **Yes** 2. **No** |  | |
| **KNOWLEDGE ASSESSEMENT** | | | | |
|  | **HAD YOU HEARD OF HEPATITIS E VIRUS (HEV) BEFORE TODAY?** | 1. **Yes** 2. **No *(jump to question …..)*** | |  |
|  | **WHERE DID YOU HEAR ABOUT HEV** | 1. **At school** 2. **Antenatal clinic** 3. **Media** 4. **Not sure** | |  |
|  | **WHERE YOU TOLD ANYTHING AT THIS ANC ABOUT HEV?** | 1. **Yes** 2. **No** | |  |
|  | **CAN A PERSON GET HEV THROUGH ANY OF THESE MEANS?** | **1= Yes 2=No 3=Don’t know**   1. **Coughing on you** 2. **Sexual intercourse** 3. **Drinking water** 4. **Foods** 5. **Skin contact** 6. **Blood transfusion** | |  |
|  | **CAN A PREGNANT WOMAN GIVE HER FOETUS (BABY) HEV** | 1. **Yes** 2. **No** 3. **Don’t know** | |  |
|  | **BEFORE TODAY WHAT CONDITION DID YOU THINK HEV USUALLY CAUSES?** | 1. **Lung disease** 2. **Breast cancer** 3. **Liver disease** 4. **Cervical cancer** 5. **Don’t Know** | |  |
|  | **HEV CAN LEAD TO ABORTION OR MISCARRAIGE?** | 1. **True** 2. **False** 3. **Don’t know** | |  |
|  | **HEV CAN LEAD TO THE DEATH OF THE PREGNANT WOMAN?** | 1. **True** 2. **False** 3. **Don’t know** | |  |
|  | **HOW CAN HEV INFECTION BE PREVENTED?** | **1= Yes 2=No 3=Don’t know**   1. **Vaccination** 2. **Cough etiquette** 3. **Bath twice a day** 4. **Food hygiene** 5. **Water hygiene** | |  |
| ***THANK YOU*** | | | | |

**APPENDIX A: QUESTIONAIRRE**

| **TSIR ASJM: Kankc, nhwehwjmu akwanhorow na akwan a yjfa do nya Hepatitis E yarba wc apemfo a wcwc Oguaa Mansin wc Cman Ghana mu.** | | | |
| --- | --- | --- | --- |
| **DATE OF INTERVIEW:** | | **STUDY CODE *(pre printed)*:** | **UNIQUE NUMBER:** |
| **NAME OF INTERVIEWER:** | | **SIGNATURE:** |
| **SOCIO-DEMOGRAPHICS & RISK FACTORS** | | | |
| **No** | **NSEMBISA** | **CODE** | **NYIANO** |
| **Q1** | **WO MFE A EDZI** | **(kyerjw mfe pckyee a edzi)** |  |
| **Q2** | **EDWUMA** | 1. **Oguadzinyi** 2. **Aban edwuma** 3. **Asopitsi edwuma** 4. **Edwuma a onhia ntsetsee biara** 5. **Mennyj edwuma** 6. **Binom** |  |
| **Q3** | **ESOM** | 1. **Kristonyi** 2. **Nkramosomyi** 3. **Abosomsomnyi** 4. **Binom** |  |
| **Q4** | **AWAR MU GYINABEW** | 1. **Mennwaree** 2. **Mawar** 3. **Metse obi nkyjn** 4. **Megyaa awar** 5. **Kunafo** |  |
| **Q5** | **NWOMASUA GYINABEW** | 1. **Mannkc skuul** 2. **Primary** 3. **J.S.S** 4. **Middle skuul Form 4** 5. **Nscwdo skuul** 6. **Esuapcn** |  |
| **Q6** | **ETSE HENFA?** | **(kyerjw kurow no dzin)** |  |
| **Q7** | **HENFA NA INYA NSU A ENOM?** | 1. **Pipe** 2. **Bula** 3. **Amansan bula** 4. **Nsutsen** 5. **Binom (kyerjw)** |  |
| **Q8** | **AFA YAFUN MPJN AHEN?** | **(kyerjw yafun dodow a afa) sj wo nyiano yj oho a kc Q10 do.** |  |
| **Q9** | **NYINSJN NO MU AHEN NA ABOFRA NO WUII WC WO YAMU?** | **(kyerjw dodow no)** |  |
| **Q10** | **NYINSJN NO MU AHEN NA IYII GUII?** | **(kyerjw dodow no)** |  |
| **Q11** | **WO MBA BAAHEN NA WCTSE ASE?** | **(kyerj dodow no)** |  |
| **Q12** | **WO HIV TSEBEA TSE DJN?** | 1. **Menya bi (+)** 2. **Minnyaa bi (–)** 3. **Minnyim** |  |
| **Q13** | **AGYE BOGYA DA ANAA?** | 1. **Nyew** 2. **Oho** |  |
|  | **NYIMDZII NHWEHWJMU** |  |  |
| **Q14** | **ATSE HEPATITIS VIRUS (HEV) DA ANSAANA NDJ ANAA?** | 1. **Nyew** 2. **Oho** |  |
| **Q15** | **HENFA NA ETSEE HEV?** | 1. **Wc skuul** 2. **Antenatal kliniki** 3. **Nsjmkyerjwfo hc** 4. **Menngye nndzi** |  |
| **Q16** | **WCKAA HEV NE ANC HO BIRIBI KYERJJ WO?** | 1. **Nyew** 2. **Oho** |  |
| **Q17** | **HEV BOTUM AFA DJM AKWANHOROW YI DO ASAA OBI ANAA?** | **1.Nyew 2. Oho 3. Minnyim**  **1. Obi bc hoa gu wo do**  **2. Mpamu nkitsahodzi**  **3. Nsunom**  **4. Edziban**  **5. Honam bedzi ehyia**  **6. Bogya twetwe** |  |
| **Q18** | **PEMFO BOTUM AMA NE BA HEV YARBA NO BI ANAA?** | 1. **Nokwar** 2. **Cnnyj nokwar** 3. **Minnyim** |  |
| **Q19** | **ANSAANA EBJBA HA NO, EBJN YARBA NA EDWEN DJ HEV DZE MA OBI?** | 1. **Menemu yarba** 2. **Enufu yarba** 3. **Berjbo yarba** 4. **Cervical cancer** 5. **Minnyim** |  |
| **Q20** | **HEV BOTUM EYI ANAA ASJJ OBI NE YAFUN ANAA?** | 1. **Nokwar** 2. **Cnnyj nokwar** 3. **Minnyim** |  |
| **Q21** | **HEV BOTUM EKUM CPEMFO NO ANAA?** | 1. **Nokwar** 2. **Cnnyj nokwar** 3. **Minnyim** |  |
| **Q22** | **YEBEYJ DJN ESIW HEV YARBA NO ANO?** | 1. **Edurfa** 2. **Yebosua kwan pa a yjbcbc woa** 3. **Yeboguar mpjn ebien dabiara** 4. **Edziban ho banbc pa** 5. **Nsu ho banbc pa** |  |

**MEDA HOM ASE.**
